# Biodegradation of Methyl *tert*-Butyl Ether by Co-Metabolism with a *Pseudomonas* sp. Strain

**DOI:** 10.3390/ijerph13090883

**Published:** 2016-09-06

**Authors:** Shanshan Li, Shan Wang, Wei Yan

**Affiliations:** Department of Environmental Science & Engineering, Xi’an Jiaotong University, Xi’an 710049, Shaanxi, China; shanshan0320@xjtu.edu.cn (S.L.); wangshanw@stu.xjtu.edu.cn (S.W.)

**Keywords:** co-metabolic degradation, *Pseudomonas* sp., MTBE, alkane

## Abstract

Co-metabolic bioremediation is supposed to be an impressive and promising approach in the elimination technology of methyl *tert*-butyl ether (MTBE), which was found to be a common pollutant worldwide in the ground or underground water in recent years. In this paper, bacterial strain DZ13 (which can co-metabolically degrade MTBE) was isolated and named as *Pseudomonas* sp. DZ13 based on the result of 16S rRNA gene sequencing analysis. Strain DZ13 could grow on *n*-alkanes (C_5_-C_8_), accompanied with the co-metabolic degradation of MTBE. Diverse *n*-alkanes with different carbon number showed a significant influence on the degradation rate of MTBE and accumulation of *tert*-butyl alcohol (TBA). When *Pseudomonas* sp. DZ13 co-metabolically degraded MTBE with *n*-pentane as the growth substrate, a higher MTBE-degrading rate (*V_max_* = 38.1 nmol/min/mg_protein_, *K_s_* = 6.8 mmol/L) and lower TBA-accumulation was observed. In the continuous degradation experiment, the removal efficiency of MTBE by *Pseudomonas* sp. Strain DZ13 did not show an obvious decrease after five times of continuous addition.

## 1. Introduction

Co-metabolism is defined as “the capability of microorganisms to transform the non-growth substrate in the presence of a growth substrate or another biodegradable substrate” [1]. It has been considered to be a prospective alternative for the elimination of pollutants, because it separates the process of biodegradation of pollutants from the growth of microorganisms, resulting in a shortening of the period of adaption and propagation. Currently, our knowledge of the clear mechanism of co-metabolism in a pure culture is limited. Some studies have indicated that microorganisms utilize the primary substrate (growth-supporting substrate) for their cell growth, and that some enzyme was induced for the transformation of the growth substrate, which can also catalyze the conversion of the non-growth substrate [2]. Co-metabolism has been confirmed in the elimination of alkane, aromatic, and chlorinated compounds in nature, including methyl *tert*-butyl ether (MTBE).

Since it was synthesized, MTBE has been used as an effective gasoline oxygenate because of its favorable properties, including low production cost, a high octane rating capability, and the ability to be miscible with other gasoline components. With the worldwide use of MTBE and increasing reports of its contamination in underground water, more concerns have been raised about the development of the technology used for its elimination [3]. Due to the presence of a stable ether bond and sterically-hindered carbon in its molecular structure, MTBE is relatively difficult to be assimilated by microorganisms [4]. Until now, only a few microorganisms were reported to have the capability of growing with MTBE as the sole carbon source, including *Methylibium petroleiphilum* PM1 [5], *Aquincola tertiaricarbonis* L108 [6], *Hydrogenophaga flava* ENV735 [7], accompanied with low growth rate and biomass yield. A variety of MTBE-degrading microorganisms could oxidize MTBE by co-metabolism in the presence of another growth substrate. Various compounds present in gasoline have been found that could be the co-metabolic growth substrates for MTBE degradation, such as normal and branched alkanes, aromatics [4,8,9]. Smith et al. reported that *Pseudomonas mendocina* KR-1 co-metabolically degraded MTBE with an average degradation rate of 61.1 nmol/min/mg_protein_ on growth of *n*-alkanes (C_5_-C_8_). Although MTBE was eliminated effectively, stoichiometric accumulation of *tert*-butyl alcohol (TBA) was observed. Two MTBE-degrading strains—*Pseudomonas aeruginosa* BM-B-450 and *Pseudomonas citronellolis* BM-B-447—were isolated from *n*-pentane-adapted consortium with different kinetic parameters for MTBE, which suggested the cooperative degradation in the consortium [4].

Though a variety of microorganisms were reported for their ability to degrade MTBE by co-metabolism, our knowledge of the co-metabolic mechanism is very limited. Smith et al. characterized the initial reaction during the co-metabolic degradation pathway of MTBE by *n*-propone-grown *Mycobacterium vaccae* JOB5, and found that an alkane hydroxylase that catalyzed the transformation of *n*-octane was also responsible for MTBE oxidation [10]. Numerous studies indicated that non-specific monooxygenase enzymes are responsible for the initial oxidation of MTBE to TBA, including cytochrome P450 monooxygenases [11,12] and alkane hydroxylase [10,13]. Furthermore, alkane hydroxylases and their close homologues appear to be widely distributed, especially among gram-negative organisms that use *n*-alkanes as growth substrates. This may help interpret why alkanes are the preferred co-metabolic substrates for the degradation of MTBE.

Therefore, the objective of this work was to isolate the single strain which could degrade MTBE co-metabolically from the consortium grown on *n*-octane. Then, we characterized the co-metabolism of MTBE by this strain, including the range of the co-metabolic substrate, the kinetic characterization, and the continuous degradation of MTBE. These results provide a basis for further MTBE elimination in the environment.

## 2. Experimental Section

### 2.1. Strains and Media

The single strain used in this study was isolated from mixed culture RS, which can co-metabolically degrade MTBE with *n*-octane as the growth substrate [14]. The mineral salt media (MSM) used for microorganism growth and MTBE degradation in this study was designed as follows (g/L): KH_2_PO_4_ 0.9, Na_2_HPO_4_·12H_2_O 6.5, (NH_4_)_2_SO_4_ 0.4, MgSO_4_·7H_2_O 0.2, CaCl_2_·2H_2_O 0.01, FeSO_4_·7H_2_O 0.001, and 1 mL of trace element solution, which was composed of the following trace elements in 1 L of deionized water: 0.1 g of H_3_BO_3_, 0.4 g of CoCl·6H_2_O, 0.25 g of ZnCl_2_, 1 g of MnSO_4_·H_2_O, 0.25 g of Na_2_MoO_4_·2H_2_O, 0.1 g of NiSO_4_·6H_2_O, and 0.25 g of CuCl_2_·2H_2_O. The pH value of the medium was adjusted to 7.0, and then the medium was autoclaved at 115 °C for 15 min. The whole-grown mixed RS culture was diluted and smeared on MSM (*n*-octane + MTBE) agar plates to screen single colonies. Various single colonies were first cultivated in lysogeny broth (LB) broth overnight and then for other analysis. Cell growth was measured by monitoring the optical density of the culture at 600 nm (OD_600_) in an iMark Microplate Reader (BioRad, Hercules, CA, USA).

### 2.2. Chemicals

All gaseous alkanes (methane, ethane, propane, *n*-butane) were purchased from Xi’an Standard Gas Station. MTBE (99%), TBA (99%), *n*-pentane (99%), *n*-hexane (99%), *n*-heptane (99%), *n*-octane (99%), and *n*-nonane (99%) were obtained from Sinopharm Chemical Reagent Co., Ltd. (Shanghai, China).

### 2.3. Identification of the Isolated Strain

The isolated single strain was identified by 16S rRNA-based sequence analysis. Genomic of isolated single strain was extracted with Ezup Column Bacteria Genomic DNA Purification Kit (Sangon Biotech, Shanghai, China) according to the manufacturer’s instructions. Partial 16S rRNA sequence was amplified from the genomic extraction with universal primers 27F (5′-AGAGTTTGATCCTGGCTCAG-3′) and 1492R (5′-GGTTACCTTGTTACGACTT-3′). The amplification reaction mixtures (50 µL) contained 1 µL of template, 1.5 U of Takara PrimeSTAR HS DNA Polymerase (Takara Biotech, Dalian, China), 10 µL of 5× PrimeSTAR buffer, 2 µL each of forward and reverse primer (10 µmol/L), and 5 µL of dNTP mixture (2.5 mmol/L). Amplifications were carried out on a T100™ Thermal Cycler (Bio-Rad) with the following conditions: 98 °C for 5 min, then 30 cycles of 98 °C for 10 s, 55 °C for 5 s, and 72 °C for 1.5 min, followed by a final extension at 72 °C for 7 min. The PCR product after purification was sent to Sangon (Sangon Biotech) for Sanger sequencing.

The DNA sequence obtained was analyzed for the most closely-matching sequence by BLAST through the GenBank database [15]. Phylogenetic analysis of the 16S rRNA sequence was performed on MEGA 5.1 software [16]. Multiple alignment with the GenBank database was performed based on Clustal method [17]. A phylogenetic tree was constructed with the neighbor-joining method.

### 2.4. The Co-Metabolic Culture Conditions of the Microorganisms

All the co-metabolic MTBE-degrading experiments were conducted in triplicate in 125 mL serum bottles sealed with Teflon Mininert valves containing 20 mL of MSM. The carbon source was added to the bottles with syringes at a concentration of 50 mg/L in the presence or absence of MTBE (10 mg/L). The bottles were incubated at 30 °C with shaking at 150 rpm. Cell growth was measured by monitoring the optical density of the culture at 600 nm (OD_600_).

The continuous degradation experiments were performed as described above. The isolated strain was cultivated in MSM with *n*-pentane (50 mg/L) in the presence of MTBE (10 mg/L). The *n*-pentane and MTBE were re-spiked as their concentrations dropped below 5% of the initial concentrations. The repeated addition was conducted four times.

### 2.5. Kinetic Analysis

For the kinetic experiments, the isolated strain was cultivated with various initial concentrations of MTBE and harvested by centrifuge at 5000 rpm for 10 min in a Sorvall™ ST 40 Centrifuge (ThermoFisher Scientific, Cambridge, MA, USA). The sediments were heated at 65 °C in 3 mmol/L of sodium hydroxide for 30 min to lyse the cells. After centrifugation, the supernatant was used to determine the concentration of total protein by Folin phenol method [18]. Bovine serum albumin (BSA) was used as a standard. The rate of MTBE oxidation was calculated from the final TBA concentration, assuming that the MTBE oxidation rate remained constant for the first 30 min, and that no further oxidation of TBA occurred during this period. The kinetic constants were derived by fitting the data by nonlinear regression to a single substrate-binding model *y* = *V*_max_·(*x*/(*K_s_* + *x*)) with Origin version 6.1 software (OriginLab, Northampton, MA, USA).

### 2.6. Analytical Method

MTBE and TBA concentration were analyzed by headspace solid-phase dynamic extraction-gas chromatography-mass spectrometry (HS-SPDE-GC/MS) method. Liquid culture was collected and centrifuged at 6000 rpm for 10 min. Two milliliters of the supernatant were added in a 10 mL glass vial and immediately sealed with a screw cap and silicone-polyperfluoroethylene gaskets for HS-SPDE analysis on a CTC Combi PAL-*xt* autosampler (Chromtech, Idstein, Germany) equipped with a polar WAX syringe (polyethylene glycol coated) as the method described previously [14]. The sample absorbed on the SPDE syringe was automatically injected into a TraceGC ULTRA (ThermoFinnigan, Milano, Italy) gas chromatograph equipped with a HP-5MS capillary column (30 m length, 0.25 mm ID, 0.25 μm film) and a TraceISQ (ThermoFinnigan, Austin, TX, USA) mass spectrometric detector. The split/splitless injection port was set in splitless mode and held at 260 °C. The oven temperature was held at 40 °C for 2 min and was ramped to 120 °C at 5 °C/min. The flow rate of carrier gas (Helium 5.0) was 1.0 mL/min. The interface and ion source temperatures were maintained at 280 °C and 230 °C, respectively. The mass spectrometry was operated in electron impact mode at 70 eV in selected ion monitoring (SIM) mode at *m*/*z* 73 and 59 for MTBE and TBA, respectively. The MTBE and TBA were quantified using standards of known concentrations.

## 3. Results and Discussion

### 3.1. The Isolation and Identification of MTBE-Cometabolic-Degrading Strain

One of the key factors which constrain the development of biodegradation of MTBE is the extremely low biomass of microorganisms grown on MTBE. The potential alternative is co-metabolism, which has been confirmed during the process of MTBE elimination. Co-metabolism describes the transformation of a non-growth substrate, when the microbes are cultivated with another compound as the growth substrate.

In order to isolate the single strain with higher biomass production on optimum growth substrate, *n*-alkanes and aromatics were tested for their ability to support the growth of the isolated strains. As shown in Table 1, all four strains could grow on the aromatic compounds tested. However, all of the isolated strains could not utilize any gaseous *n*-alkanes (C_1_ to C_4_), but grow well on the liquid *n*-alkanes tested (C_5_ to C_8_) with a much higher biomass. Among the four single strains, strain DZ13 showed the maximum biomass concentration cultivated with *n*-alkanes. Furthermore, the final biomass production of DZ13 on different *n*-alkanes decreased with the length of carbon chain of *n*-alkanes. MTBE or TBA could not be utilized as the growth substrate of strain DZ13.

**Figure 1:**
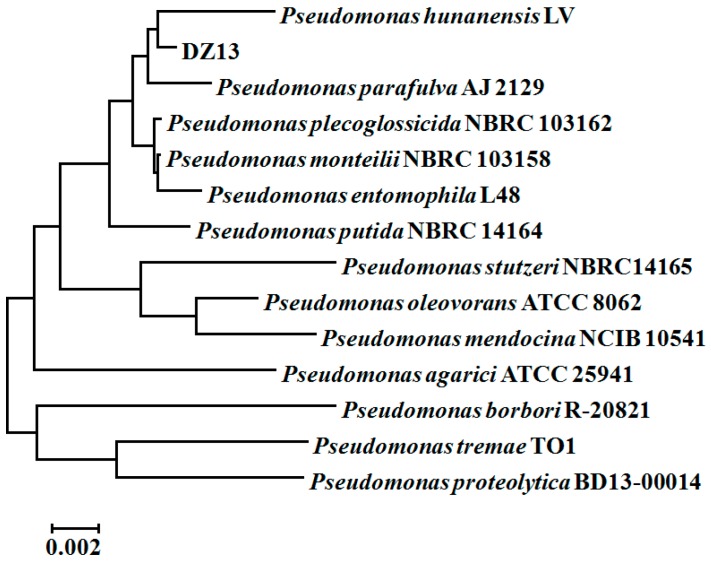
Phylogenetic tree of *Pseudomonas* sp. DZ13 based on 16S rRNA sequences. The tree is constructed by the neighbor-joining method of the bootstrap test (1000 replicates). The tree is drawn to scale, with branch lengths in the same units as those of the evolutionary distances used to infer the phylogenetic tree. All positions containing gaps and missing data were eliminated from the dataset (Complete deletion option).

Finally, the pure strain DZ13, which was gram-negative and short rod shaped, was selected for further study. The 16S rRNA-based sequence analysis revealed that strain DZ13 belongs to the *Pseudomonas* genus, and was named *Pseudomonas* sp. DZ13. *Pseudomonas hunanensis* LV was found to be phylogenetically closest to the isolated strain DZ13, showing 99% sequence similarity between the 16S rRNA genes (Figure 1). The 16S rRNA sequence of the isolated DZ13 strain was deposited in NCBI GenBank database under the accession number of KX301316.

According to previous reports, members of the *Pseudomonas* genus have been found in numerous environmental niches on Earth and have demonstrated the ability to mineralize various pollutants, such as polycyclic aromatic hydrocarbon (PAH) [19,20], benzene [21], alcohols [22,23], etc. The various desirable characteristics of *Pseudomonas* genus strains make them meet the requirements of industrial biotechnology, including higher biomass productions and lower nutritional demands. Furthermore, numerous typical strains from the genus *Pseudomonas* have been whole-genome sequenced, including *P. aeruginosa* PAO1, *P. putida* KT2440, and *P. mendocina* NBRC 14162. The broad substrate scope and easy accessibility of genetic bioinformation facilitate the further utilization of *Pseudomonas* genus strains in biotechnology and bioremediation.

### 3.2. Co-Metabolic Degradation of MTBE with Different n-Alkanes as Growth Substrate

Co-metabolism, which has extended the range of degrading substrates by microorganisms, has been confirmed in many pollutant degradation processes, including chlorinated compounds [24,25], aromatic hydrocarbons [26,27] and gasoline additives [28], including MTBE [8,11].

Various studies have identified that microorganisms can co-metabolically degrade MTBE efficiently when cultured with a variety of growth substrates, such as alkanes, aromatics, and alicyclics [8,29]. Our results bring some interesting insights into the co-metabolism of MTBE by *Pseudomonas* sp. DZ13. First of all, some kinds of *n*-alkanes and aromatics could be utilized by strain DZ13 as the carbon source. Though strain DZ13 was isolated from mixed culture inoculated with *n*-octane as the sole carbon source, other *n*-alkanes and aromatics were tested for their capability to support the growth of DZ13 and the degradation of MTBE (Table 1). For the aromatic compounds tested, *Pseudomonas* sp. DZ13 could grow on toluene and xylene (low biomass yield), accompanied with the slow degradation of MTBE. All of the *n*-alkanes tested in this study (C_5_-C_8_) could support the growth of *Pseudomonas* sp. DZ13 with a much higher degradation rate than that of aromatic compounds. As the carbon number of the *n*-alkanes increased, both the degradation rate of *n*-alkanes and MTBE (from 178.4 mg_MTBE_/g_protein_/h to 59.3 mg_MTBE_/g_protein_/h) were decreased. These results were similar to the reports for MTBE co-metabolism by Garnier et al. [30]. All *n*-alkanes (*n*-pentane, *n*-hexane, and *n*-heptane) tested for co-metabolic activity did support the degradation of MTBE. Among them, *n*-pentane was the most efficient, with a degradation rate of (200 µg/d). In another study, the co-metabolic degradation rate of MTBE by *P. mendocina* KR-1 increased with the carbon number of the *n*-alkanes used as the growth substrate [31].

Another piece of evidence worth highlighting was that TBA could be further oxidized by *Pseudomonas* sp. DZ13 during the co-metabolism of MTBE. After two days’ cultivation, low levels of TBA accumulation (less than 7%) were observed for all the *n*-alkanes tested (Table 2). The simplest interpretation of this phenomenon is that strain DZ13 could synthesize the enzyme which can catalyze the oxidation of TBA. This result was similar to that reported by Morales et al. [4]. The process of MTBE degradation by *Pseudomonas aeruginosa* showed no TBA accumulation when the initial ratio of MTBE and *n*-pentane was below 0.7. However, for another reported strain (*P. mendocina* KR-1), although MTBE was transformed with a high co-metabolic degradation rate (61.1 nmol/min/mg_protein_), the corresponding accumulation of TBA was observed for all *n*-alkanes tested (C_5_ to C_8_), which indicated the absence of the enzyme catalyzing the transformation of TBA in strain KR-1. Neither MTBE nor TBA could be utilized as the growth substrate of *Pseudomonas* sp. DZ13.

The co-metabolic coefficient (CC) was used to represent the co-metabolic efficiency. The CC value is defined as the ratio of degraded MTBE to the consumed co-metabolic substrate [4]. The lowest and highest CC values of 0.03 and 0.72 for *Pseudomonas* sp. DZ13 was obtained from the growth substrates toluene and *n*-pentane, respectively (Table 2). The lower CC value for aromatic substrates was compatible with the lower degradation rate of MTBE with different alkanes. When cultivated with *n*-alkanes, the CC value of *Pseudomonas* sp. DZ13 decreased with increasing carbon number of the growth substrate. The shorter-chain alkane (which was more soluble than the longer one) could be transferred to the microorganisms more quickly, which may be the potential reason that the CC value was higher for the shorter-chain alkane than for the longer one.

As shown in Table 2, a positive correlation between the co-metabolic biodegradation rate of MTBE and the specific oxidation rate of growth substrate was observed. In other words, the degradation rate of MTBE raised with the increasing oxidation rate of the corresponding substrate. This result indicates that the oxidation of MTBE as the growth substrate may be catalyzed by the same enzyme. This finding was similar to that of Tran et al. [32].

### 3.3. The Kinetic Characteristics of the Co-Metabolism of MTBE by Pseudomonas *sp.* DZ13 on n-Alkane

**Figure 2:**
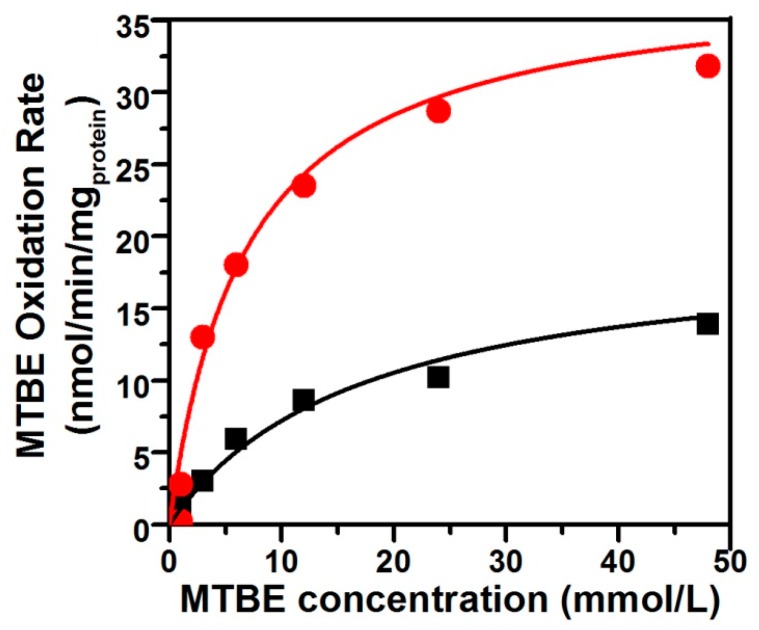
Kinetics of MTBE oxidation by pure strain DZ13 with *n*-pentane and *n*-octane as co-metabolic substrates. The figure shows the average rate of MTBE oxidation by strain DZ13 on *n*-pentane (filled red circles) or *n*-octane (filled black squares). All of the experiments were performed in triplicate.

The oxidation of TBA—the first stable metabolite detected in MTBE-degrading pathway—is the limiting step of the mineralization of MTBE. The apparent TBA accumulation during the first period of MTBE oxidation by *n*-alkane-grown cells indicated that the kinetics of MTBE oxidation could be directly determined by measuring the rates of TBA accumulation. Values of *K_s_* and *V_max_* for MTBE oxidation were determined for pure DZ13 culture grown on *n*-pentane and *n*-octane, respectively, in the presence of MTBE (Figure 2). When cultivated with the equivalent concentration of MTBE and *n*-alkane with different carbon number (C_5_ and C_8_) on the same condition, *Pseudomonas* sp. DZ13 showed different kinetic parameters. The values for *K_s_* of 6.8 mmol and 17.2 mmol, for *V_max_* of 38.1 nmol/min/mg_protein_ and 19.6 nmol/min/mg_protein_ were determined for *Pseudomonas* sp. DZ13 grown on *n*-pentane and *n*-octane, respectively. The alternative explanation of this phenomenon may be the different aqueous solubility of each *n*-alkane. The Henry’s Law constants of *n*-pentane and *n*-octane were 8.1 × 10^−4^ M/atm and 3.14 × 10^−4^ M/atm (at 25 °C), respectively [33]. When incubated with the same initial concentration of *n*-pentane or *n*-octane, the higher dissolved quantity of *n*-pentane facilitated its capture by the DZ13 cells and then induced the transcription of alkane monooxygenase, resulting in the degradation of *n*-pentane and MTBE.

Another interesting piece of evidence was that the *K_s_* value for MTBE determined for strain DZ13 in this study was much higher than those of co-metabolically MTBE-degrading microorganisms on the growth substrate of *n*-alkanes. For instance, the *K_s_* value for MTBE obtained from *Arthrobacter* sp. ATCC27778 [34] on butane was 24 µmol, and the *n*-alkane-oxidizing *P. mendocina* KR-1 has a *K_s_* value of 12.95 µmol for MTBE [31]. The simplest interpretation of this result is that variable alkane monooxygenase responsible for the transformation of MTBE are involved in different strains. Specific affinity (a°_MTBE_ = *V_max_*/*K_s_*) is generally used as an index reflecting substrate specificity. The value for *Pseudomonas* sp. DZ13 (a°_MTBE_ = 0.34 L/g_protein_/h) on *n*-pentane was almost four times higher than the value on *n*-octane (a°_MTBE_ = 0.07 L/g_protein_/h), mainly due to the large difference between *K_s_* values. Similar a°_MTBE_ values were obtained with *P. mendocina* KR-1 (0.28 L/g_protein_/h), which also has a higher *K_s_* value [31]. Though higher a°_MTBE_ values were calculated for *Xanthobacter* sp. (1.27 L/g_protein_/h) and *M. vaccae* JOB5 (1.08 L/g_protein_/h) [35], TBA accumulation was observed accompanying the MTBE degradation process.

### 3.4. Degradation of MTBE by Pseudomonas *sp.* DZ13 Grown on n-Pentane in Continuous Culture

**Figure 3:**
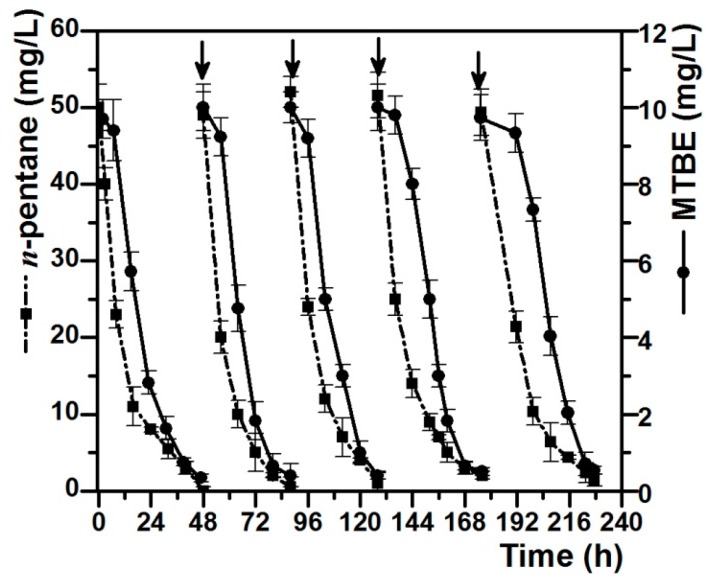
Co-metabolic degradation of MTBE with *n*-pentane as growth substrate. Vertical arrows indicate the additions of MTBE and *n*-pentane. MTBE (10 mg/L) and *n*-pentane (50 mg/L) were re-spiked when the residual concentrations of both MTBE and *n*-pentane in the cultures were below 5% of the initial concentrations. All of the values are the means of triplicate.

Further research on the co-metabolic degradation of MTBE with *n*-pentane as the growth substrate was conducted to determine the continuous degradation ability of strain DZ13. MTBE (10 mg/L) and *n*-pentane (50 mg/L) were re-spiked when the residual concentrations of both MTBE and *n*-pentane in the cultures were below 5% of the initial concentrations. Figure 3 showed the degradation curves of MTBE and *n*-pentane after repeated feeding and degrading for five times. Pure strain DZ13 can co-metabolically degrade MTBE (10 mg/L) at the rate of 45.3–52.5 mg_MTBE_/g_protein_/h with *n*-octane (50 mg/L) as the growth substrate. An obvious lag phase was observed in the MTBE-degradation curve compared to the *n*-octane-degradation curve, in which *n*-pentane was transformed immediately once it was added. The first degradation circle of MTBE and *n*-octane was completed in two days. After being re-spiked, *n*-pentane was degraded continuously, accompanied by the removal of MTBE. It has been observed that the MTBE degradation rate did not apparently decrease after the fifth addition.

These results suggest that the pure strain *Pseudomonas* sp. DZ13 had the co-metabolic capability for the continuous degradation of MTBE when cultivated with *n*-pentane as the growth substrate. As reported by Fortin et al., a mixed F-consortium could degrade MTBE in the presence or after incubation of diethyl-ether (DiEE) after two re-spikes. MTBE would only be transformed after the complete degradation of the co-metabolic carbon source, which indicated that MTBE biodegradation undergoes some kind of inhibition [36]. Volpe et al. inoculated a microbial consortium in a batch reactor with repeated feeding of MTBE and oxygen. After several rounds of feeding and depletion, a significant decrease in the time course of complete degradation of MTBE was observed [29].

MTBE was most often detected in the field of gasoline-contaminated soil or groundwater, accompanied by the detection of other gasoline components (such as alkanes), which is confirmed as the growth substrates of co-metabolically MTBE-degrading microorganisms. Various studies were conducted to complete the MTBE elimination efficiently and quickly. The results of this paper show the desirable perspectives for the bioremediation of MTBE by co-metabolism.

## 4. Conclusions

The results of this study indicated that single strain DZ13, which belonged to *Pseudomonas* sp., demonstrated the ability to degrade MTBE by co-metabolism on *n*-alkane growth substrates (C_5_-C_8_). The degradation rate of MTBE by strain DZ13 varied with the *n*-alkane growth substrate carbon number. *Pseudomonas* sp. DZ13 could co-metabolically degrade more than 95% of MTBE within two days with extremely low TBA residue, and the removal efficiency of MTBE did not show obvious decrease after five times of continuous addition. This research is expected to provide a reference for the development of a MTBE-remediation technique.

## Figures and Tables

**Table 1:** The cell growth of the different single strains on potential growth substrates.

Potential Growth Substrates ^a^	OD_600_ for 5 Days Cultivation ^b^			
	DZ2	DZ6	DZ9	DZ13
*n*-Alkanes				
Methane	<0.01	<0.01	<0.01	<0.01
Ethane	<0.01	<0.01	<0.01	<0.01
Propane	<0.01	<0.01	<0.01	<0.01
*n*-Butane	<0.01	<0.01	<0.01	<0.01
*n*-Pentane	0.086 ± 0.003	0.132 ± 0.019	0.192 ± 0.018	0.285 ± 0.013
*n*-Hexane	0.124 ± 0.007	0.196 ± 0.033	0.227 ± 0.024	0.238 ± 0.010
*n*-Heptane	0.136 ± 0.018	0.123 ± 0.006	0.175 ± 0.016	0.211 ± 0.013
*n*-Octane	0.179 ± 0.021	0.145 ± 0.013	0.184 ± 0.026	0.175 ± 0.026
Aromatics				
Benzene	0.086 ± 0.011	0.045 ± 0.004	0.062 ± 0.006	0.022 ± 0.008
Toluene	0.075 ± 0.017	0.031 ± 0.008	0.095 ± 0.004	0.074 ± 0.013
*o*-Xylene	0.096 ± 0.006	0.051 ± 0.006	0.033 ± 0.007	0.094 ± 0.006
*p*-Xylene	0.114 ± 0.021	0.062 ± 0.016	0.038 ± 0.009	0.088 ± 0.018
*m*-Xylene	0.089 ± 0.014	0.057 ± 0.012	0.045 ± 0.003	0.082 ± 0.003
MTBE and its metabolites				
MTBE	<0.01	0.012 ± 0.005	<0.01	<0.01
TBA	<0.01	<0.01	<0.01	<0.01

**^a^** Triplicate cultures of each single strains were grown for 5 days in the presence of each substrate at an initial substrate concentration of 0.02% (*v*/*v*) (The initial value of OD_600_ was 0.01); **^b^** All optical densities reported are the means ± SD. MTBE: methyl *tert*-butyl ether; OD_600_: optical density at 600 nm; TBA: *tert*-butyl alcohol.

**Table 2:** Specific degradation rates and products by *Pseudomonas* sp. DZ13 on diverse substrates.

Carbon Source ^a^	Biomass (mg_protein_/d)	Degradation Rate of Substrates ^b^ (mg_substrate_/g_protein_/h)	Degradation Rate of MTBE ^b^ (mg_MTBE_/g_protein_/h)	CC (mg_MTBE_/mg_substrate_)	Residual TBA ^c^ (mg/L)
Aromatic substrate					
Benzene	0.3 ± 0.1	3.6 ± 0.2	0	-	-
Toluene	8.3 ± 0.7	247.3 ± 7.9	4.6 ± 0.1	0.03 ± 0.01	0.12 ± 0.06
Xylene	9.5 ± 0.5	435.8 ± 10.5	12.3 ± 0.9	0.11 ± 0.06	1.34 ± 0.1
*n*-Alkanes					
*n*-Pentane	15.2 ± 1.1	1393.2 ± 78.1	178.4 ± 8.4	0.72 ± 0.2	0.14 ± 0.1
*n*-Hexane	14.6 ± 1.4	1096.7 ± 56.4	130.2 ± 9.2	0.56 ± 0.1	0.56 ± 0.1
*n*-Heptane	12.2 ± 0.4	841.7 ± 53.9	72.3 ± 7.1	0.44 ± 0.1	0.67 ± 0.1
*n*-Octane	10.9 ± 0.8	681.6 ± 45.8	59.3 ± 6.9	0.26 ± 0.1	0.26 ± 0.1
Others					
MTBE	-	-	-	-	-
TBA	-	-	-	-	9.8 ± 0.3

**^a^** *Pseudomonas* sp. DZ13 was cultivated in glass serum vials (125 mL) containing mineral salt medium (20 mL) on diverse carbon source (0.02% *w/v*) in the presence (20 μmol) of MTBE. After two days’ growth, the biomass concentrations were examined. All of the cultures on diverse carbon sources were performed in triplicate. All of the values were the means of triplicate ±SD; **^b^** After two days’ cultivation, the amounts of residual carbon source and MTBE in each culture were determined by solid-phase dynamic extraction-gas chromatography-mass spectrometry (SPDE-GC-MS). The degradation rate of growth substrate and MTBE were calculated, respectively; **^c^** After two days’ cultivation, the amount of TBA produced in each culture was determined by SPDE-GC-MS. CC: co-metabolic coefficient.
